# Assessment of short and long-term outcomes of diabetes patient education using the health education impact questionnaire (HeiQ)

**DOI:** 10.1186/s13104-017-2536-6

**Published:** 2017-06-15

**Authors:** Ditte Hjorth Laursen, Karl Bang Christensen, Ulla Christensen, Anne Frølich

**Affiliations:** 1 Research Unit of Chronic Conditions, Bispebjerg and Frederiksberg Hospital, Bispebjerg Bakke 23, 20D, 2400 Copenhagen NV, Denmark; 2 Department of Biostatistics, Institute of Public Health, University of Copenhagen, Øster Farimagsgade 5, Postboks 2099, 1014 Copenhagen K, Denmark; 3 Department of Social Medicine, Institute of Public Health, University of Copenhagen, Øster Farimagsgade 5, Postboks 2099, 1014 Copenhagen K, Denmark

**Keywords:** Patient education, Type 2 diabetes, Health education impact questionnaire, Self-management, Denmark

## Abstract

**Background:**

Type 2 diabetes is a progressive chronic illness that will affect more than 500 million people worldwide by 2030. It is a significant cause of morbidity and mortality. Finding the right care management for diabetes patients is necessary to effectively address the growing population of affected individuals and escalating costs. Patient education is one option for improving patient self-management. However, there are large discrepancies in the outcomes of such programs and long-term data are lacking. We assessed the short and long-term outcomes of diabetes patient education using the health education impact questionnaire (HeiQ).

**Methods:**

We conducted a observational cohort study of 83 type 2 diabetes patients participating in patient education programs in Denmark. The seven-scale HeiQ was completed by telephone interview at baseline and 2 weeks (76 participants, 93%) and 12 months (66, 80%) after the patient education ended. Changes over time were assessed using mean values and standard deviation at each time point and Cohen effect sizes.

**Results:**

Patients reported improvements 2 weeks after the program ended in 4 of 7 constructs: skills and technique acquisition (ES = 0.59), self-monitoring and insight (ES = 0.52), constructive attitudes and approaches (ES = 0.43) and social integration and support (ES = 0.27). After 12 months, patients reported improvements in 3 of 7 constructs: skills and technique acquisition (ES = 0.66), constructive attitudes and approaches (ES = 0.43), and emotional wellbeing (ES = 0.44). Skills and technique showed the largest short- and long-term effect size. No significant changes were found in health-related activity or positive and active engagement in life over time.

**Conclusion:**

After 12 months, diabetes patients who participated in patient education demonstrated increased self-management skills, improved acceptance of their chronic illness and decreased negative emotional response to their disease. Applying HeiQ as an outcome measure yielded new knowledge as to what patients with diabetes can obtain by participating in a patient education.

## Background

An estimated 500 million people will be diagnosed with type 2 diabetes mellitus (T2DM) by 2030, and diabetes prevalence will continue to rise [1, 2]. Affected individuals have a mortality rate twice that of the general population, and many patients are at significant risk of developing diabetes-related complications such as myocardial infarction, stroke, nephropathy, retinopathy and peripheral arterial disease and neuropathy resulting in amputation [3, 4]. T2DM is a major health issue in Denmark and by 2040 it is estimated that every 6th will have diabetes [5]. Effective self-management by patients is an important part of diabetes care and a crucial element in effectively addressing the growing population with diabetes and escalating costs of care. Structured patient education programs are an important part of care, teaching patients how to use disease-specific self-care skills. These skills include monitoring and managing symptoms, adhering to treatment regimes, maintaining a healthy lifestyle and managing the impact of the illness on daily functioning, emotions and social relationships [6].

Self-management of blood glucose has long been considered a mainstay of diabetes self-management [7]. A review of diabetes self-management programs showed that 86% of included studies used HbA1c as an outcome measure [8]. Although HbA1c is a highly important indicator for diabetes patients, there is only indirect evidence that self-management programs are associated with modest improvements in HbA1c [9]. Instead, other outcome measurements have been used to evaluate the effect of patient education, such as health status (SF36) [10, 11], health-related quality of life [10, 12] diabetes complications [4], self-monitoring of blood glucose [11] and a range of clinical outcomes [13]. The majority of evaluations found that patient education has a positive effect on several indicators [4], but that effect generally decreases or vanishes over time [14]. Few diabetes self-management studies assessed outcomes over a period longer than 12 months, and, among those that did, many supported the conclusion that intervention benefits cannot be maintained over the long term [8]. However, outcome measures previously used to assess the effect of patient education may lack the ability to detect long-term effects. New outcome measures are warranted.

Effective self-management enables patients to ‘monitor one’s condition and to effect the cognitive, behavioral and emotional responses necessary to maintain a satisfactory quality of life’ [15]. The health education impact questionnaire (HeiQ) is designed to measure the effectiveness of health education programs based on patients’ perspectives [16]. Founded on a range of chronic conditions such as arthritis, hypertension, anxiety or depression, asthma, injury, diabetes and heart disease, the HeiQ offers a new approach to measuring cognitive, behavioral and emotional responses and fills an important gap in patient-centered outcome assessment of patient education. Studies using the HeiQ found that it captured different aspects than did standard measures typically used to assess the effect of patient education [17]. Studies further concluded that HeiQ constructs are valid and reliable measures of key dimensions of generic health-related behavior and may advance outcome assessment by also serving as goals of self-management programs [17, 18].

The aim of this study is to assess the applicability of the health education impact questionnaire (HeiQ) and to describe short and long-term outcomes of diabetes patient education.

## Methods

### Study design

An observations cohort study was conducted among T2DM patients participating in patient education in the Capital Region of Denmark. Questionnaires were administered three times: 2 weeks before patient education started (baseline, T1) and 2 weeks (T2) and 12 months (T3) after it ended.

### Patient education programs

Patients with diabetes in the Capital Region of Denmark are treated according to a regional T2DM disease management program, which are provided at most municipalities and outpatient clinics [19, 20]. When patients are diagnosed with T2DM, they are referred to a standardized rehabilitation program that aims to support them in living a healthier life with their disease. The program includes disease-specific patient education, dietary counselling, advice about physical activity and smoking cessation support, which has been described in the regional diabetes patient education guidelines [20]. The objectives of diabetes patient education are to support informed decision-making, self-care behaviors, problem-solving and active collaboration with the health care team and to improve clinical outcomes, health status and quality of life [20]. These objectives are accomplished through health professional-provided education in group sessions 1–2 times per week over 2–10 weeks.

At the time of data collection (2011), group-based diabetes patient education programs were offered in 14 of 29 municipalities and 5 of 9 hospitals in the Capital Region of Denmark. Only patient education programs offering at least 10 h of education were included in the study to maximize the likelihood of capturing any impact on participants’ health-related behavior; 5 municipalities and 2 hospitals were included. Although all hospitals and municipalities follow the same standardized patient education guidelines [20], programs varied slightly. Three programs offered additional physical activity in combination with education, 2 included weekly weight assessments, and 2 included voluntary cooking lessons (Table 1).

**Table 1:** Description of the included patient education programs

Patient education locations	Number of weeks	Hours per week	Total number of hours	Max. number of participants	Physical training	Weekly weight assessment	Cooking lessons
Municipality 1	10	2	20	20	X	X	X
Municipality 2	7	1.5	10.5	10	X	X	
Municipality 3	7	3.5	24.5	16	X		X
Municipality 4	6	3	18	14			
Municipality 5	4	3	12	16			
Hospital 1	5	2	10	12			
Hospital 2	2	6	12	16			

A letter providing information about the project was sent to all participants enrolled in the included programs and was followed a few days later by a telephone call. Participants were asked to complete the baseline and subsequent questionnaires by telephone interviews that lasted approximately 15 min. While answering the questionnaire, participants also added comments and explained their responses; this information was included as background material to provide a better understanding of participants.

**Fig. 1:**
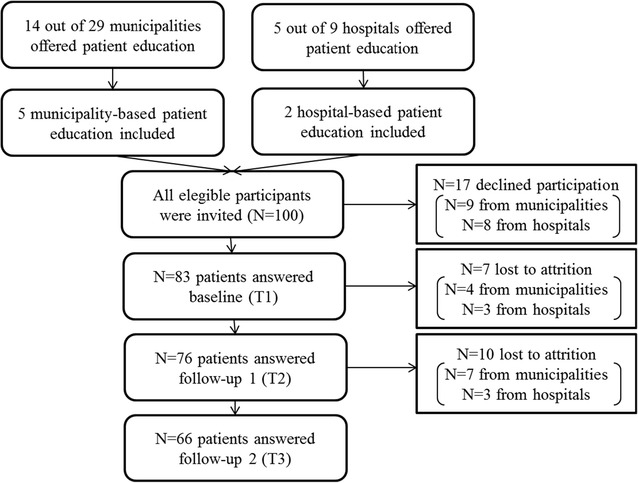
Flowchart of included participants at baseline (T1), first follow-up (T2) and second follow-up (T3)

A total of 100 individuals were invited to participate in the study (Fig. 1). Reasons for declining participation included lack of time or interest; individuals who could not be contacted by phone were excluded after 4 attempts to reach them at varying times between 8 a.m. and 9 p.m.

The following variables were measured at baseline: sex, age, marital status, cohabitation status, parental status, level of primary school education, vocational training, occupational status and self-rated health. Disease-specific variables included in the questionnaire included duration of diabetes, other chronic diseases, hypertension, high cholesterol, smoking status and body mass index (BMI).

### The health education impact questionnaire

The HeiQ is a patient-developed questionnaire designed to measure the effectiveness of patient education programs. The dimensions covered by the questionnaire target the areas that patients, clinicians, health educators, policymakers and researchers regard as crucial outcomes of patient education programs for people with chronic disease [16]. Thus, the HeiQ is a generic, patient-reported outcome measure used across settings and disease groups. It consists of 35 items across 7 independent constructs: health-directed activity; positive and active engagement in life; emotional wellbeing; self-monitoring and insight; constructive attitudes and approaches; skill and technique acquisition; social integration and support (Table 2). Each construct comprises an independent questionnaire, and all constructs collectively provide a comprehensive profile of the intended outcomes of health education [16]. Each construct-specific questionnaire includes 4–6 items rated on a 4-point scale (1 = strongly disagree, 2 = disagree, 3 = agree, 4 = strongly agree). The sum of scores for all items is divided by the number of items; construct scores range between 1 and 4. A higher score indicates better self-management, with the exception of the emotional wellbeing construct for which scoring is reversed. The questionnaire has demonstrated high content and face validity, resulting from the grounded process by which constructs and items were derived. Strong evidence of construct validity was established using a rigorous confirmatory factor analysis [16].

**Table 2:** Description of the 7 HeiQ constructs and Cronbach’s alpha

Construct	Description	Cronbach’s alpha
Health-directed activity	Functional disease prevention and/or health promotion lifestyle activity	0.57
Positive and active engagement in life	Motivation to be active and involved in life, including behavioural elements, such as participation in life activities, and psychological elements, such as enthusiasm for life activities	0.67
Self-monitoring and insight	Insight into living with a health problem, including how individuals engage in self-monitoring of health problems, their acknowledgement of realistic illness-related limitations and ability and confidence to adhere to these limitations	0.51
Constructive attitudes and approaches	An attitude held by individuals that they are not going to let health problems control their lives, including how individuals view the impact of their condition on their life	0.76
Skill and technique acquisition	Improvement in knowledge-based skills and techniques to manage health	0.66
Social integration and support	Positive impact of social engagement and support that evolves through interaction with others and includes the confidence to seek support from interpersonal relationships, as well as from community-based organizations	0.83
Emotional wellbeing	Negative affect, such as anxiety, stress, anger and depression	0.76

The HeiQ was translated into Danish using the WHO translational framework [21] in the following way: (1) forward translation from English into Danish by 2 independent translators; (2) reconciliation in which 2 researchers came to a consensus on the draft of the Danish translation of the HeiQ that best reflected the literal and conceptual content of the original English HeiQ; (3) backward translation of the Danish version into English by 2 professional English translators who were not familiar with the original English version of the HeiQ; and (4) backward translation review and finalization in which the original HeiQ developer and researchers reviewed the backward translation against the source instrument and ensured the literal and conceptual equivalence of the translation. The HeiQ was then culturally adapted by the first author [22]. Pilot interviews with 10 participants across patient education programs verified that questions were generally understood as intended.

Cronbach’s alpha [23] was computed for each construct to estimate internal consistency (reliability); values for constructs ranged from 0.51 to 0.83 (Table 2). We further evaluated fit of each item to the Rasch model [24–26], using a comparison of observed and expected item-rest score correlation [27]. No evidence of misfit was seen (results not shown).

### Statistical analysis

We computed the mean and standard deviation (SD) for the 7 HeiQ constructs at each point in time, the mean and 95% confidence interval (CI) for changes in scores between baseline and T2 and between baseline and T3. Change scores were further evaluated as Cohen effect sizes (ES; change scores standardized using the pooled baseline SD) to assess the magnitude of changes in scores between baseline and each subsequent point in time. We considered ES = 0.1, ES = 0.3 and ES = 0.5 to indicate small, medium and large changes, respectively. We used Chi squared tests to evaluate if baseline covariates differed significantly for drop-outs. SAS 9.4 was used for all analyses.

### Ethical approval

The study was approved by the Danish Data Protection Agency (number: BBH-2011-08 Diabetespatientuddannelse). Under Danish law, permission from an ethics committee was not required because biological material was not used in the study.

## Results

Of 100 subjects invited to participate in the study, 83 completed the baseline questionnaire. Of these, 76 provided follow-up data at T2 (93%) and 66 provided follow-up data at T3 (80%). We found borderline significant differences in the proportion of participants who dropped out between T1 and T2 for age (p = 0.03), with those younger than 65 years and older than 75 years dropping out. Participants who dropped out between T1 and T3 were significantly more likely to be younger than 65 or older than 75 (p = 0.01) or employed (p = 0.04) and have low cholesterol (p = 0.03) (results not shown).

Table 3 shows baseline demographic characteristics for participants. Of the 83 participants, 52% were males. More than half were under 65 years of age (55%) and 60% were married. The largest proportion of participants had been diagnosed with diabetes between 1 and 10 years (49%, mean = 6, 6 years). Eighty percent reported hypertension and 78% had high cholesterol, while half reported having another chronic condition in addition to diabetes (46%). Nearly half of all participants (47%) self-rated their health as good, and only 4% rated their health as poor. Just 17% of participants were of normal weight. Overall, data reflect a diverse population; despite a high rate of obesity, hypertension and high cholesterol, the largest proportion of participants still rated their health as good.

**Table 3:** Participant characteristics, n (%)

	Participants (n = 83)
Male	43 (52)
Age, years	
<65	46 (55)
65–75	30 (36)
76+	7 (8)
Marital status	
Married/living with a partner	50 (60)
Widowed/divorced/separated	33 (40)
Cohabitation status	
Living alone	35 (42)
Living with spouse and/or children	48 (58)
Level of primary school	
Elementary school	66 (80)
High school	17 (20)
Education beyond primary school	
None	10 (12)
Vocational training	48 (58)
Short higher education	6 (7)
Intermediate higher education	13 (16)
Long higher education	6 (7)
Occupational status	
Employed	19 (23)
Unemployed	64 (77)
Self-rated health	
Excellent	5 (6)
Very good	16 (19)
Good	39 (47)
Less good	20 (24)
Poor	3 (4)
Duration of diabetes (years)	
<1	24 (29)
1–10	41 (49)
>10	18 (22)
Hypertension	66 (80)
High cholesterol	65 (78)
Current smokers	13 (16)
BMI	
Normal weight	14 (17)
Overweight	25 (30)
Obese	25 (30)
Severely obese	19 (23)

### T2: short-term changes in HeiQ constructs

Two weeks after patient education ended and compared to baseline values, significant improvement and a large effect size were observed in the skills and technique acquisition construct (ES = 0.59, p < 0.001) and self-monitoring and insight (ES = 0.52, p < 0.001) (Table 4). Medium effect sizes were found in one constructs: constructive attitude and approaches (ES = 0.43, p < 0.001), while social integration and support only showed small significant changes (ES = 0.27, p = 0.003).

**Table 4:** Changes in HeiQ constructs over time

	T1 (N = 83)		T2 (N = 76)		T3 (N = 66)		From T1 to T2			From T1 to T3		
	Mean	SD	Mean	SD	Mean	SD	Change, 95% CI	p value	ES	Change, 95% CI	p value	ES
Health-directed activity	2.96	0.72	3.07	0.76	3.08	0.90	0.11, −0.04 to 0.25	0.16	0.15	0.12, −0.02 to 0.25	0.08	0.16
Positive and active engagement in life	3.42	0.51	3.49	0.49	3.53	0.55	0.06, −0.03 to 0.16	0.18	0.12	0.09, −0.04 to 0.21	0.17	0.17
Emotional wellbeing	2.94	0.67	3.12	0.73	3.26	0.86	0.18, 0.04 to 0.32	0.01	0.26	0.30, 0.10 to 0.49	0.004	0.44
Self-monitoring and insight	3.23	0.47	3.45	0.37	3.36	0.59	0.25, 0.15 to 0.35	<0.001	0.52	0.10, −0.05 to 0.26	0.18	0.22
Constructive attitudes and approaches	3.51	0.47	3.71	0.35	3.71	0.44	0.20, 0.10 to 0.31	<0.001	0.43	0.20, 0.09 to 0.31	<0.001	0.43
Skill and technique acquisition	2.94	0.59	3.28	0.67	3.33	0.73	0.35, 0.19 to 0.52	<0.001	0.59	0.40, 0.20 to 0.59	<0.001	0.66
Social integration and support	3.30	0.65	3.44	0.65	3.44	0.65	0.18, 0.06 to 0.29	0.003	0.27	0.12, −0.03 to 0.26	0.11	0.18

*CI* confidence interval, *ES* effect size, *M* mean, *N* number, *SD* standard deviation, *SRH* self-reported health, *T2* 2 weeks, *T3* 12 months

### T3: long-term changes in HeiQ constructs

Twelve months after patient education ended and compared to baseline values, a large effect size was observed in skills and technique acquisition (ES = 0.66, p < 0.001) (Table 4). Statistically significant medium effect sizes were observed in constructive attitude and approaches (ES = 0.43, p < 0.001) and emotional wellbeing (ES = 0.44, p = 0.004). Even though social integration and support showed improvements at T2, the improvement was not re-found at T3. Emotional wellbeing did, however, show medium improvements after 12 months (ES = 0.44, p < 0.01), although only small improvements were seen over the short term.

Only two constructs demonstrated to maintain positive improvement over both the short- and long-term periods: skills and technique acquisition and constructive attitude.

## Discussion

This study assessed short and long-term outcomes of T2DM patient education programs in the Capital Region of Denmark using the HeiQ as the outcome measure. Improvement was seen in skills and technique acquisition and constructive attitude and approaches over both short- and long-term measurement periods. After 12 months, emotional wellbeing also showed significant improvements, as compared to baseline scores.

The large effect size in the construct of skill and technique acquisition at both 2 weeks and 12 months (0.59 and 0.66, respectively) represents the largest gain for participants in patient education programs. The construct aims to capture change in knowledge-based skills and techniques (including the use of equipment) that participants acquire or re-learn to help them manage their disease-related symptoms and health problems [16]. Patient education for diabetes patients involves significant emphasis on how patients should correctly and regularly measure their glucose level and interpret other test results. In fact, in most diabetes literature, self-management generally refers to patient mastery of technical skills, such as home glucose monitoring [13]. In the patient education programs included in the study, health professionals presented blood glucose monitoring devices to patients and taught them how to perform measurements. Patients were also taught skills pertaining to diet and exercise and new ways of incorporating this knowledge into their everyday lives. The very high effect sizes in this construct demonstrate that patients gained new skills to help them better manage their disease. These results are confirmed in other studies that utilized the HeiQ [17, 28, 29]. However, it was expected that these skills would also be applied to increase participants’ level of physical activity. This was not the case. No significant improvements were seen in the construct of health-related activity over either the short or long term. These results emphasize the importance to diabetes patients of learning the right skills and also demonstrate that these skills relate primarily to technical aids, a finding that is confirmed by a Swedish study [29].

The construct of constructive attitude and approaches showed significant improvement over the short and long term. This construct is embodied in the statement “I am not going to let this disease control my life” and can detect a shift in how individuals view the impact of their condition(s) on their lives [16]. In essence, it captures acceptance of chronic illness [17]. In our study, the effect size did not change over time, indicating that acceptance of disease may be a stable characteristic among participants. This is supported by two other studies showing that people with a chronic disease develop their personal models in the early stage of the illness and that, unless challenged directly, i.e. by treatment changes, they are likely to be constant [30, 31].

The construct of emotional wellbeing showed a relatively high effect size 12 months after the intervention ended, indicating a low level of emotional wellbeing among participants. This construct measures negative affective responses to illness, including anxiety, anger and depression [16]. Previous findings have shown that patients experience a great deal of anger and anxiety shortly after diagnosis [32]. Most participants in the study reported here had had diabetes for a number of years, so it is very likely that they developed over time good self-management skills through trial and error, life experience and interaction with health-care providers [33]. Mastering their diabetes could be reflected in emotional improved wellbeing. Maunsell et al. explored the construct of emotional wellbeing in the cancer setting and found that it was associated with empowerment and very important to cancer patients [18]. To gain a deeper understanding of this construct and its applicability to patients with diabetes, it would be interesting to compare the HeiQ with the problem areas in diabetes (PAID) questionnaire, which also measures diabetes-specific emotional wellbeing [34].

### Applicability of the HeiQ in a diabetes setting

Schuler et al. explored the HeiQ and found that it both captured different aspects than did other standard outcome measures and corresponds to defined goals of self-management programs: in particular, empowerment (health-directed activity), self-management (skills and technique) and acceptance of chronic illness (constructive attitude) [17]. A study by Maunsell et al. supported these findings, showing that HeiQ constructs can be used as a generic measure of health-related empowerment [18].

Corbin and Strauss have described a range of components that are faced by people with a chronic condition who require day-to-day management after having participated in self-management education [13, 35]. When combining these with the goals defined by the American Diabetes Association [36] these tasks relate to what is being measured with the HeiQ.

1. *Clinical content and skills: medical management of the condition such as taking medication, changing diet or self*-*monitoring blood sugar*, which can be measured by the skills and technique acquisition and self-monitoring and insight constructs.
2. *Behavioural strategies: goal setting and problem solving and healthy lifestyle choices*, which can be measured with health directed activity.
3. *Engagement with psychosocial concerns: coping with the anger, fear, frustration and sadness of having a chronic condition as well as creating and maintaining new meaningful life roles regarding jobs, family and friends*. These can be measured by the social integration and support construct, emotional wellbeing and constructive attitude and approaches constructs.

These components encompass many important issues that have been identified as important for patient outcomes and six of the seven HeiQ constructs seem useful in evaluating the effectiveness of patient education if they are linked to these components.

Twelve months after patient education ended, participants reported that they had established the fundamental skills and technique to manage a chronic disease, learned to accept the disease and reduced negative emotional responses to their disease. These results provide a foundation for a new way of measuring the effectiveness of diabetes patient education programs. Although many other studies found that the effect measured by HbA1c or other clinical measures decreases or vanishes over time, we provided evidence of long-term outcomes of T2DM patient education programs in the Capital Region of Denmark. Applying the HeiQ in a diabetes setting provided new knowledge about benefits patients with diabetes can garner by participating in a patient education.

### Strength and limitations

A strength of this study is the use of telephone interviews for data collection. Although they were very time consuming, interviews provided us with a nearly complete data set for each participant at all 3 points in time. In general, telephone interviews results in more complete data, compared to mailed questionnaires [37], which was confirmed in our study, which had very few missing data. However, extreme responses in self-assessment questions are generally more frequent in telephone interviews than in mailed questionnaires [37], which may explain the higher baseline mean values for the constructs in our study, compared with other studies using mailed HeiQ questionnaires [18, 33, 38, 39]. This may indicate that our results reflect a slight overestimate. Furthermore, because participants reported high baseline scores in the HeiQ constructs, changes in some constructs may not have been adequately measured for participants with relatively little subjective disease burden, due to a ceiling effect [33].

The HeiQ is still a relatively new questionnaire; a literature search resulted in only 28 papers that were directly related to the instrument. Ongoing research is therefore needed to fully understand the applicability of the HeiQ across disease groups and settings. Further studies are also warranted to compare the HeiQ to other patient-reported outcome measures to fully understand its content and capabilities. Although no clinical data were collected in the study reported here, it would have been interesting to investigate a possible association between improved skills and techniques with HbA1c levels.

The promising results of this study indicate that patient education in Denmark actually improves patient outcomes, but the methodology was limited by the lack of a control group.

## Conclusion

This study assessed short and long-term outcomes of T2DM patient education programs using the HeiQ as the outcome measure. When engaging in T2DM patient education participants increase their self-management skills, learn to accept having a chronic illness and reduce negative emotional responses to their disease. Although many other studies found that the effect of patient education measured by clinical measures decreases or vanishes over time, this study provides evidence of long-term outcomes of T2DM patient education when outcomes are based on patients’ reported outcomes. Applying the HeiQ as an outcome measure yielded new knowledge as to what patients with diabetes can obtain by participating in a patient education.
